# The Influence of Interpersonal Patterns on the Therapy Process in a Case of Childhood Trauma

**DOI:** 10.5334/pb.511

**Published:** 2020-10-26

**Authors:** Kimberly Van Nieuwenhove, Reitske Meganck, Emma Acke, Shana Cornelis, Mattias Desmet

**Affiliations:** 1 Department of Psychoanalysis and Clinical Consulting, Ghent University, Ghent, BE

**Keywords:** childhood trauma, interpersonal dynamics, psychodynamic therapy, single case study, therapy process and outcome

## Abstract

Research concerning the influence of core interpersonal patterns related to childhood trauma on the therapeutic process is scarce. We investigated interpersonal patterns at the start of treatment, changes in interpersonal patterns as treatment progressed, and the change process in a mixed-methods single case study of a supportive-expressive psychodynamic psychotherapy with a 33-year-old female with a history of childhood trauma. The patient showed a pervasive inability to open up towards others throughout the entire treatment, which is closely associated with others’ actual or anticipated rejection, disrespect and disinterest. Excessive use of expressive interventions, which target interpersonal change, initially led to a worsening of the patient’s condition. Via supportive and general interventions, symptom stabilization was achieved. The findings of this study suggest a thorough understanding of dominant interpersonal patterns is necessary to recognize their influence on the therapy process.

People with a history of complex trauma (i.e., being exposed to prolonged and repeated interpersonal traumatic events) suffer from a wide variety of symptoms, including interpersonal difficulties (e.g., Herman, 1992; van der Kolk et al., 2005; Van Nieuwenhove & Meganck, 2017). These interpersonal problems are often related to a lack of trust in others and the world as a result of childhood experiences in which primary caregivers were unreliable and unpredictable. Drawing from attachment theory, this insecure basis gives rise to certain deeply engrained interpersonal patterns that mark interpersonal relations later in life (Pearlman & Courtois, 2005). These interpersonal patterns can be broadly defined as a seemingly coherent representational frame via which a person perceives him/herself, in relation to others and the world. This frame corresponds with the internal working model in attachment theory (Bretherton & Munholland, 2008), cognitive schemas in Piaget’s developmental theory (Wadsworth, 2004) and the Core Conflictual Relationship Theme (CCRT) in psychodynamic psychotherapy (Luborsky, 1984). The CCRT operationalizes interpersonal patterns by defining the subjective wishes with which one enters interpersonal relations (W), one’s own personal appraisal of how the other interacts and responds to these wishes (RO) and the characteristic reactions of the self to this other (RS). Previous studies, using the CCRT paradigm, have found that complex trauma is associated with the perception that others are rejecting, opposing, controlling, and – overall – bad (Chance, Bakeman, Kaslow, Farber, & Burge-Callaway, 2000; Drapeau & Perry, 2009). This interpersonal pattern, comprising a fundamental distrust toward others, is translated to a variety of interpersonal relations later in life, including the relationship with a therapist (Gleiser et al., 2008; Pearlman & Courtois, 2005).

Following the clinical predicament of the therapeutic relationship being marked by a fundamental distrust, several researchers propose a stabilization phase in the treatment of complex trauma, in which the focus primarily lies on the formation of a safe therapeutic relationship (e.g., Cloitre et al., 2012; Herman, 1992). After more than three decades of research in this area, however, the necessity of the implementation of such an initial stabilization phase remains highly controversial, with studies supporting the inclusion of a stabilization phase (e.g., Classen, Muller, Field, Clark, & Stern, 2017; Cloitre et al., 2010; Gleiser, Ford, & Fosha, 2008) and others resisting the necessity of a stabilization phase in treatment (e.g., De Jongh et al., 2016; Resick et al., 2012; Wagenmans, Van Minnen Sleijpen, and De Jongh, 2018).

Van Nieuwenhove and Meganck (2017) argue that the impasse regarding the necessity of a stabilization phase will not likely be resolved by approaching it with classical methods, such as cross-sectional comparison studies and dismantling studies. In their current form, these typical effectiveness studies only allow general causal statements (i.e., the specific treatment produces changes) and not statements about the mechanisms underlying the changes (Kazdin, 2007). Consequently, a more thorough investigation of therapy processes via N = 1 case study research is necessary to make advancements (Kazdin, 2007). Moreover, to arrive at a more comprehensive understanding, it is necessary to study some of the core assumptions underlying the need for initial stabilization. Certain questions such as if and how interpersonal patterns influence the therapeutic relationship, how therapeutic interventions can foster or hamper the establishment of a safe working alliance, and which therapeutic techniques are necessary to accomplish therapeutic change, remain unanswered.

In order to refine theory and enhance our understanding of these basic mechanisms (Levitt et al., 2018; Stiles, 2013), we aim at an in-depth investigation of interpersonal patterns in a systematic mixed-methods single case study of a woman with a background of childhood trauma. We opted for an exploratory N = 1 case study design because it allows an in-depth scrutiny of the unfolding of interpersonal dynamics in a treatment context, therefore also allowing to study their influence on the therapy process. Moreover, it allows to investigate the process of change in-depth by systematically monitoring the therapeutic relationship and therapist interventions and mapping possible shifts throughout treatment (Fishman & Messer, 2013; Stiles, 2013).

Specifically, we will study interpersonal patterns and processes in a single case of manualized supportive-expressive psychodynamic treatment, in which interpersonal patterns are targeted through supportive and expressive techniques. Concisely, supportive interventions aim to foster the therapeutic relationship by expressing the engagement to help the patient and providing an empathic and safe atmosphere. Expressive interventions, on the other hand, include clarifications and interpretations to recognize, understand and work through core interpersonal patterns, which are generally considered to be directly associated with symptoms and therefore warrant change (Luborsky, 1986). The manual of Luborsky also includes specific guidelines for working with more severely distressed patients to strengthen the therapeutic alliance by applying a greater amount of supportive interventions. As therapy progresses, and the relationship is safe enough to tolerate expressiveness, more expressive interventions can be introduced.

In summary, the first aim of this case study is to investigate the nature of interpersonal patterns in childhood trauma. Second, we will study the way early interpersonal patterns change throughout treatment. Third and finally, we will examine this process of change via a systematic study of the therapeutic alliance and therapist interventions.

## Method

### Participants

#### Client

Pam, a White female, is 33 years old the moment she entered therapy. She has a history of childhood physical and psychological abuse perpetrated by her mother, while her father remained a passive witness. According to DSM-IV-TR criteria (APA, 2000), Pam received the diagnosis of recurrent seasonal Major Depressive Disorder (MDD), agoraphobia and Body Dysmorphic Disorder. She has been taking antidepressant and anti-epileptic medication for over a decade and has been hospitalized for three months because of suicidal ideations three years prior to treatment. In order to guarantee confidentiality, we used a pseudonym. Moreover, all information that would lead to the identification of the patient has been removed or anonymized. Ethics committee approval was granted by the Ghent University Hospital (B670201523446) (Meganck et al., 2017).

#### Therapist

The therapist is a White female, who was 32 years old and had 8 years of clinical experience when therapy started. She is formally trained in Psychoanalytic Therapy and received an additional training in Short Term Psychodynamic Psychotherapy (STPP, Leichsenring & Schauenburg, 2014; Luborsky, 1984). The therapy consisted of 20 weekly sessions of STPP. Session duration ranged between 35 and 68 minutes (*M* = 51.24 minutes).

#### Case Selection

We drew our data from the Ghent Psychotherapy Study (GPS, Meganck et al., 2017), a Randomized Controlled Trial in which patients either receive 16 to 20 sessions of Cognitive Behavioral Therapy (CBT) or STPP for the treatment of MDD. Only the measures used in this study are mentioned (for a full description, see Meganck et al., 2017). We selected the case of Pam, without knowledge of outcome, using two criteria. The first requirement was the presence of a complex traumatic background (i.e., repeated and prolonged interpersonal traumatic events) as reported in the Clinical Diagnostic Interview (CDI, Westen, 2006). During the CDI, Pam describes having had a poor upbringing with a very ‘tyrannical’ mother, whom would be very controlling (e.g., regular room inspection not allowing any secrets), demanding (e.g., cleaning and cooking) and punishing (e.g., physical abuse, psychological games). The second requirement was that Pam received STPP to ensure treatment focuses on interpersonal themes. As our research objectives mainly require rich information on interpersonal dynamics, we did not set any further (diagnostic) requirements.

### Measures

#### Interview and Qualitative Measures

*The Clinical Diagnostic Interview* (CDI, Westen, 2006) is a semi-structured narrative-based interview that assesses a broad range of intra- and interpersonal characteristics. This interview allows for an in-depth understanding of important past and current relationships that appear in the story of the patient (e.g., ‘How would you describe your relationship with your mother/father/partner/…?’ or ‘Can you describe a specific situation or confrontation with him/her that typifies your relationship?’). *The Structured Clinical Interview for DSM-IV-TR* (SCID) is a structured interview to determine DSM-IV-TR axis I disorders (SCID-I, First, Spitzer, Gibbon, & Williams, 2002) and DSM-IV-TR axis II personality disorders (SCID-II, First, Gibbon, Spitzer, Williams, & Benjamin, 1997). The *Client Change Interview* (CCI, Elliott, Slatick, & Urman, 2001) is a semi-structured interview assessing the experience of the therapeutic process and therapeutic change. In the context of the GPS, the therapist joined in bi-weekly group *supervision*, in which she discussed the case of Pam two times. All interviews, therapy sessions – with the exception of session 13 where the audio recorder failed – and supervision sessions, were audiotaped and transcribed using pre-set standards.

#### Quantitative Measures

*The Beck Depression Inventory* (BDI-II, Beck, Steer, & Brown, 1996) is a 21-item self-report questionnaire used to assess depression severity. *The Self-rating Inventory for Post-Traumatic Stress Disorder* (ZIL, Hovens, Bramsen, & van der Ploeg, 2000) is a 22-item self-report questionnaire used to assess symptoms related to PTSD. *The Inventory of Interpersonal Problems* (IIP-32, Horowitz, Alden, Wiggins, & Pincus, 2000) is a 32 items self-report questionnaire used to assess interpersonal functioning on eight scales (i.e., domineering, vindictive, cold/distant, socially inhibited, nonassertive, overly accommodating, self-sacrificing, and intrusive). The *Symptom Checklist* (SCL-90-R, Derogatis, 1992) is a 90-items self-report questionnaire administered to assess general psychical and physical well-being. The *Working Alliance Inventory-Short Revised* (WAI-SR, Horvath & Greenberg, 1989) is a 12-item self-report questionnaire to evaluate the quality of the therapeutic relationship by assessing feelings of mutual trust (bond scale), consensus on treatment objectives (goals scale) and consensus about treatment implementation (task scale).

### Procedures

**Figure 1:**
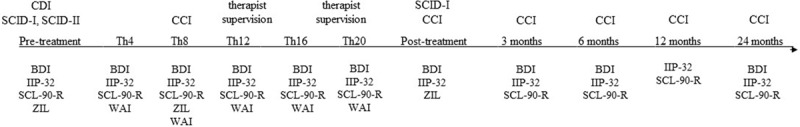
Quantitative self-report (lower half) and interview and qualitative measures (upper half) throughout the research and therapy process. *Note*: Due to missing values, the total scores for the SCL-90-R at post-treatment and the BDI-II at 12-month follow-up could not be calculated. CDI: Clinical Diagnostic Interview; SCID-I: Structured Clinical interview for DSM-IV axis-I disorders; SCID-II: Structured Clinical Interview for DSM-IV personality disorders; CCI: Clinical Change Interview; BDI: Beck Depression Inventory; IIP-32: Inventory of Interpersonal Problems; SCL-90-R: Symptom Checklist; ZIL: Self-rating Inventory for Post-Traumatic Stress Disorder; WAI: Working Alliance Inventory.

We executed an *integrative mixed-methods design* (Levitt et al., 2018) and applied principles of *Consensual Qualitative Research for case studies* (CQR-c, Jackson, Chui, & Hill, 2011), in which consensus and triangulation (i.e., using a combination of different research methods) are essential, to systematically examine interpersonal features and processes. Specifically, we used triangulation of both quantitative and qualitative measures (self-report questionnaires, interviews, therapy sessions), methods (outcome assessment, qualitative analysis, standardized coding systems) and researchers (consensus procedures, audits). See Figure 1 for a comprehensive overview of the different measures.

The *Core Conflictual Relationship Theme method* (CCRT, Luborsky & Crits-Christoph, 1998) is a manualized procedure to map dominant interpersonal patterns in narrative material derived from transcribed therapy sessions and consists of two broad steps. First, relationship episodes (REs) are selected within the narrative material, i.e., excerpts in which an interpersonal exchange is described. Second, these REs are coded to map the dominant wish (W), the (anticipated) response of the other person involved (RO) and the person’s own reaction (RS), using standard categories (Edition 2) provided by the CCRT manual (Luborsky & Crits-Christoph, 1998), which include 35 Ws, 30 ROs and 31 RSs. The CCRT method was conducted by the first and fourth author on narratives derived from the transcribed therapy sessions at the beginning (sessions 1 through 4), middle (sessions 9 through 12) and end (sessions 17 through 20) of treatment. Consensus on the frequency of each component was achieved through detailed discussion and the final frequency with which each category occurred across the REs was computed to provide the dominant CCRTs.

The *Penn Adherence/Competence Scale for Supportive-Expressive Dynamic Psychotherapy* (PACS-SE, Barber & Crits-Christoph, 1996) is a 45-item rating-scale to assess the frequency of different therapeutic techniques. The scale consists of nine items assessing general techniques, which can be broadly defined as neutral questions or comments to facilitate patient’s speech, nine items assessing supportive techniques, such as positive appraisals and an empathic conveyance of understanding and acceptance, and 27 items assessing expressive interventions, including questions to gain information on interpersonal dynamics and interpretations or statements to focus attention on or give feedback about core interpersonal patterns. All therapist’s interventions – except ‘mhm’, which was excluded from analyses for pragmatic reasons – were rated as general, supportive or expressive by the first and third author. Through consecutive meetings, consensus was achieved and the frequencies per technique were computed for every session.

## Results

### Symptoms and Outcome Assessment

At the beginning of therapy, Pam had a BDI-II score of 36, indicating *severe* depressive complaints (Beck et al., 1996). Her IIP-32 score of 57 indicates interpersonal problems are *above average*. Her scores on the subscales of the IIP suggest *significant* difficulties with being ‘socially inhibited’, ‘non-assertive’ and ‘overly accommodating’ and *above average* difficulties with being ‘cold/distant’ and ‘self-sacrificing’ (Horowitz et al., 2000). Her SCL-90-R score of 231 indicates *very high* overall symptom burden (Derogatis, 1992).

**Figure 2:**
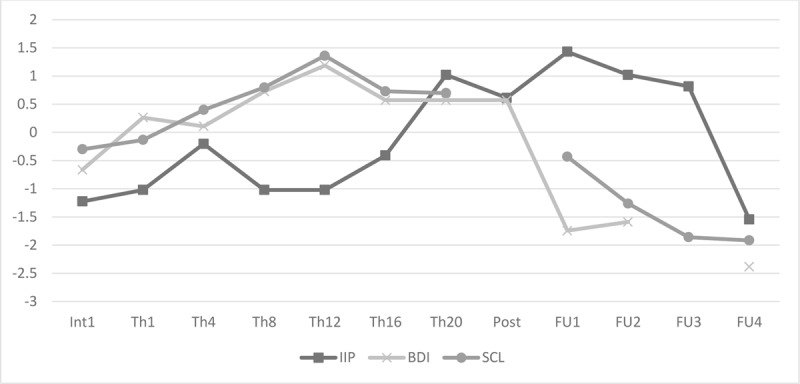
Evolution in outcome measures (z-scores). *Note*: IIP: Inventory of Interpersonal Problems; BDI: Beck Depression Inventory; SCL-90-R: Symptom Checklist.

As Figure 2 illustrates, her scores on the outcome measures continue to increase as treatment progresses and remain high at the end of treatment. Her scores suggest *severe* depression (BDI-II = 44, Beck et al., 1996), *significant* interpersonal difficulties (IIP-32 = 68, Horowitz et al., 2000) and an overall *very high* symptom burden (SCL-90-R=261, Derogatis, 1992). At the end of treatment, her scores on the IIP-32 subscales ‘socially inhibited’ and ‘non-assertive’ are *above average* and her scores on the subscales ‘cold/distant’, ‘overly accommodating’ and ‘self-sacrificing’ suggest *significant* difficulties in these areas (Horowitz et al., 2000). When assessed with the Reliable Change Index (RCI, Jacobson & Truax, 1991), the increasing trend indicates a clinically significant deterioration on the SCL-90-R (RCI = 1.966, >1.96, p < .05) and no change on the BDI-II (RCI = 1.678, p > .05) and the IIP-32 (RCI = 1.647, p > .05). Taking the cut-off of 52 into account (Hovens et al., 2009), Pam’s scores on the ZIL indicate that she was suffering from symptoms related to PTSD both before (ZIL = 59), during (as measured before session 8, ZIL = 70) and after (ZIL = 61) treatment, despite not meeting the basic criteria for the SCID-diagnosis of PTSD. Albeit the pre-post scores suggest therapy failure, the qualitative analysis from the CCIs warrants some nuance. At the end of treatment, Pam noticed a remarkable change in her sentiment and vigour, which has not been captured in the self-report questionnaires. Noteworthily, she said: “I feel worse on paper than I actually do.” Also, the SCID-I, which was conducted by an independent researcher after treatment termination, revealed no indications of MDD. After treatment termination, Pam’s scores on the outcome measures show a decreasing trend and two years after treatment ended clinical significant improvement was achieved on the BDI-II (RCI = –4.61, <–1.96, p < .05) and her ZIL-score of 46 dropped below the cut-off of 52 (Hovens et al., 2009).

### CCRT Analysis

Table 1 shows the dominant CCRT components for phase 1 (sessions 1 through 4), phase 2 (sessions 9 through 12) and phase 3 (sessions 17 through 20). For each phase, we will describe the most prominent CCRT components and illustrate them with excerpts from the REs derived from the corresponding therapy sessions.

**Table 1:** The dominant wish (W), response other (RO) and response self (RS) throughout therapy.

	#	W	RO	RS

Phase 1	9	to avoid conflict (9)/ to not be responsible or obligated (4)/ to assert myself (4)/ to be respected (3)/ to be helped (3)/ to not be hurt (3)/ to be accepted (2)/ to be my own person (2)/ to be loved (2)	are rejecting (8)/ are controlling (5)/ are not understanding (3)/ dislike me (3)/ are distant (3)/ are bad (3)/ don’t respect me (2)/ are not trustworthy (2)/ are unhelpful (2)/ hurt me (2)/ oppose me (2)/ are angry (2)	am not open (9)/ feel anxious (7)/ am dependent (6)/ feel angry (6)/ dislike others (3)/ am helpless (3)/ am out of control (2)/feel depressed (2)/ feel guilty (2)
Phase 2	12	to avoid conflict (10)/to be respected (6)/ to be accepted (5)/ to be open (3)/ to be loved (3)/ to be liked (2)/ to not be hurt (2)/ to not be responsible or obligated (2)	don’t respect me (5)/ are rejecting (5)/ are not understanding (4)/ are not trustworthy (4)/ are distant (4)/ are strong (3)/ are controlling (2)	am not open (10)/ am helpless (5)/ am uncertain (5)/ feel angry (5)/ feel anxious (5)/ am dependent (4)/ feel disappointed (4)/ feel unloved (3)
Phase 3	12	to be respected (8)/ to have trust (8)/ to be accepted (6)/ to be liked (6)/ to be understood (4)/ to be opened up to (4)/ to be open (4)/ to be helped (3)/ to not be hurt (3)/ to be loved (2)	are rejecting (8)/ are controlling (7)/ don’t respect me (5)/ are distant (5)/ are strong (5)/ are not understanding (4)/ are not trustworthy (4)/ are strict (4)/ are unhelpful (3)/ are accepting (2)/ respect me (2)	am not open (8)/ feel disappointed (8)/ oppose others (5)/ am dependent (5)/ am helpless (4)/ don’t understand (3)/ dislike others (3)/ feel self-confident (3)/ am uncertain (3)/ feel angry (3)/ am self-controlled (2)/ feel unloved (2)/ feel anxious (2)

*Note*: #: amount of RE’s, W: the dominant wish, RO: response other, RS: response self, (x) amount of RE’s in which the CCRT component occurs.

In the first phase, all REs center on the wish to avoid conflict in relation to others. Especially in relation to her parents (3 REs), Pam experiences a lot of criticism (RO ‘are rejecting’). She wants to break free from them (W ‘to not be responsible or obligated’) and wishes to be recognized in her own right (W ‘to assert myself’, ‘to be respected’). However she says nothing (RS ‘am not open’) and passively undergoes (RS ‘am dependent’) their intimidation and domination (RO ‘are controlling’) out of fear (RS ‘am anxious’) and to protect herself (W ‘to not be hurt’, ‘to avoid conflict’).

P: They just show up unannounced and walk in without asking if it suits me or not (RO ‘are controlling’). I don’t speak up (RS ‘am not open’) when something bothers me. I do not dare to say (RS ‘am anxious’) that it does not work out well for me at that moment. I’m so annoyed by it (RS ‘am angry’). I feel like a slave (RS ‘dependent’). They say all sorts of negative things, sometimes pure criticism (RO ‘are rejecting’), for instance that it is not clean enough. I don’t react. I don’t go into discussion with them (W ‘to avoid conflict’). I do not set any limits. I would want to (W ‘to assert myself’), but towards my parents, I just can’t do it (RS ‘am helpless’).

This pattern is also clearly shown in relation to others in her life, both in relation to her husband (2 REs) and in work-related contacts (4 REs). The next RE concerning her husband illustrates how she does not open up because of the anticipated reaction, rather than his actual reaction:

P: If I think about it, I know that I don’t have to be afraid (RS ‘feel anxious’) for questions he might ask. He means well. But still, the idea that he might say things such as ‘you don’t do anything around the house’ (RO ‘are rejecting’), makes me not talk about it (RS ‘am not open’).

Between sessions 9 through 12, the dominant CCRT components do not particularly change. At large, this can be explained by the fact that seven REs concern interactions with her parents, which show a very rigid pattern. However, it seems that another layer of her core interpersonal issues got unraveled in this phase. Table 1 shows that in the negative reaction of others, next to the critical and controlling demeaner (RO ‘are rejecting’, ‘are controlling’), more emphasis is placed on the fact that people do not value her or treat her fairly (RO ‘don’t respect me’), are unsympathetic and inconsiderate (RO ‘are not understanding’) and unresponsive or unavailable (RO ‘are distant’). In the same respect, the wish to be affirmed (W ‘to be accepted’), to be important to others (RO ‘to be respected’) and others to show an interest in her (W ‘to be liked’) prevail. Parallel to the first phase, we see similarities in the relation between Pam and her parents (7 REs) and her husband (2 REs):

P: My parents don’t ask (RO ‘are distant’), so I keep silent (RS ‘am not open’). I have the idea that it just does not interest them (RO ‘don’t respect me’, ‘dislike me’). They don’t ask and I’m not going to talk spontaneously about how that was for me (W ‘to be respected’, ‘to be liked’). It seems as if they don’t care, so… yeah.

P: I think my husband knows by now that the relation with my parents is a difficult topic for me, but how and what exactly, he does not know. He does not ask anything about it, so… (RO ‘are distant’, ‘are not understanding’).

In this phase, Pam does express the wish to be able to be more open towards others, especially her sister (2 REs) (e.g. ‘I would like to be able to open up to people that are close to me.’). However, she experiences a strong ambivalence (RS ‘am uncertain’) and inability to do so (RS ‘am helpless’).

At the end of therapy, in line with the first two phases, the reactions of others are perceived or anticipated in a negative way (e.g. RO ‘are rejecting’, ‘don’t respect me’, ‘are controlling’). In this phase, Pam mainly talks about interactions with people she perceives as having an authoritarian position (e.g., parents, doctors, bosses). She discloses how she would always remain silent (RS ‘am not open’) and would passively submit to their superiority (RS ‘am dependent’, RO ‘are strong’, RO ‘are controlling’) whilst feeling disappointed (RS ‘feel disappointed’) about their negligence (RO ‘are not understanding’, ‘don’t respect me’, ‘are distant’) and denunciation (RO ‘are rejecting’, ‘are not trustworthy’). In this phase, the wishes show a notable shift. She emphasizes wanting others to be sincerely interested (W ‘to be respected’, ‘to have trust’, ‘to be liked’) in who she really is (W ‘to be accepted’, ‘to be understood’). Moreover, she wants to be able to have genuine conversations (W ‘to be opened up to’, ‘to be open’). Whereas before, her wishes were formulated in terms of wanting to avoid the negative anticipated reactions of others, she now seems to articulate her own desire, stemming from what she misses in relation to others.

### Therapeutic Alliance

Pam’s scores on the WAI-SR subscales, measured after the fourth therapy session (on a scale of 1 to 5, task scale = 4.5, goal scale = 4, bond scale = 4.25), suggest that feelings of mutual trust and consensus on treatment objectives were established early in treatment (Stinckens et al., 2009). Her scores show a slight decrease towards session 12 (task scale = 4, goal scale = 3.25, bond scale: 3.75) and remain stable or increase again towards the end of treatment (task scale = 4, goal scale = 3.5, bond scale = 4.5). Overall, these scores suggest that a good therapeutic relationship was formed at the start of treatment, which remained quite stable throughout the entire therapy process (Stinckens et al., 2009).

In the CCI after session 8, Pam describes her therapist as a professional and friendly person. She explains how she finds it comforting that the therapist asks questions when she experiences difficulties to come up with topics to talk about. After treatment termination, Pam recounts the therapist felt familiar and safe. If she would ever consider to go back to therapy, she would return to her because of the therapist’s professional attitude, the fact that she asked the right questions and their good connection.

### Therapist Interventions

**Figure 3:**
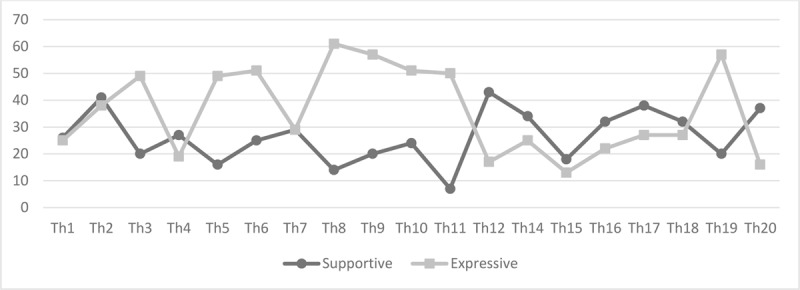
Supportive and expressive interventions throughout the treatment.

Table 2 shows the total distribution of supportive, expressive and general interventions throughout therapy. Over 19 sessions (session 13 not included) there were a total of 2,495 interventions (*M* = 119, SD =22). On average, there were significantly more general (*M* = 69, SD = 18) than expressive (*M* = 36, SD = 16, t(18) = 7.83, p < .001) and supportive (*M* = 26, SD = 10, t(18) = 10.21, p < .001) techniques per session. Over the course of treatment, expressive techniques were used significantly more, on average, than supportive interventions (t(18)= 2.68, p < .05). Figure 3 shows the evolution in the amount of supportive and expressive techniques per session. In the first two sessions, there was a higher number of supportive techniques. Between sessions 3 and 11, the number of expressive techniques was higher, whereas between sessions 12 and 18 the opposite is true. Expressive interventions show a peak in session 19 and there is a higher rate of supportive interventions in session 20.

**Table 2:** The frequency of supportive, expressive and general interventions per session.

	Duration	General	Supportive	Expressive	Total

Th1	55’33	94 (65)	26 (18)	25 (17)	145
Th2	46’22	60 (43)	41 (29)	38 (27)	139
Th3	55’35	82 (54)	20 (13)	49 (32)	151
Th4	41’54	52 (53)	27 (28)	19 (19)	98
Th5	59’12	111 (63)	16 (9)	49 (28)	176
Th6	49’00	83 (52)	25 (16)	51 (32)	159
Th7	50’26	62 (52)	29 (24)	29 (24)	120
Th8	49’42	47 (39)	14 (11)	61 (50)	122
Th9	57’41	52 (40)	20 (16)	57 (44)	129
Th10	67’43	88 (54)	24 (15)	51 (31)	163
Th11	52’40	74 (56)	7 (5)	50 (38)	131
Th12	42’05	42 (41)	43 (42)	17 (17)	102
Th14	51’00	68 (54)	34 (27)	25 (20)	127
Th15	49’00	76 (71)	18 (17)	13 (12)	107
Th16	50’35	69 (56)	32 (26)	22 (18)	123
Th17	49’59	69 (51)	38 (28)	27 (20)	134
Th18	53’43	78 (57)	32 (23)	27 (20)	137
Th19	56’22	61 (44)	20 (14)	57 (41)	138
Th20	34’59	41 (44)	37 (39)	16 (17)	94
Total		503 (20)	683 (27)	1309 (52)	2495

*Note*: (xx): percentage of total interventions.

There are, on average, 119 interventions each session, which comes down to more than 2 interventions every minute. This means that the interventions follow each other in rapid succession. Most of these interventions are general interventions, with a percentage of 39 to 71 of all interventions, in which the therapist repeats small phrases or asks neutral questions to allow Pam to elaborate on a certain situation or feeling. Pam does not spontaneously talk in great length about anything, whether or not it concerns intimate or difficult topics, requiring a more active stance from the therapist. As treatment progresses, the amount and content of general techniques remain stable with only a slight decreasing trend, which implies that Pam remained rather reticent to talk spontaneously up until the end of therapy.

In absolute numbers, supportive techniques are marginally more prevalent than expressive techniques and are used throughout the first two session. At the end of these sessions, supportive techniques are more stacked and convey a commitment from the therapist to work together.

T: I find it really important that you talk and that *we* explore *together* what is going on and what is important to you. […] *We* will take our time to figure things out *together*.

Expressive techniques incline between sessions 1 and 3 and mostly concern questions to gather information about Pam’s relationships, especially with regards to not being able to open up and the very tense relationship with her mother.

P: I have never understood and I guess I never will…………. And I don’t know if I even want to know./T: How do you mean?/P: I have been asked before if I didn’t want to know why my mother reacts the way she does, but frankly, I really don’t need to gain insight in those people. No./T: As if gaining an understanding would be equal to wiping things out./P: Yes./T: Do you have the idea that your story would disappear?/P: No… What happened in the past stays and… I don’t need. No, I just don’t need to specialize myself in my mother’s behaviour./T: Some people say okay, I want to understand because I don’t want to end up with the idea that she didn’t love me, that it had to do with something else./P: … Yeah, I don’t know what to think of it./T: You really don’t have a clue as to why she was so cold towards you?/P: … … … I don’t know if she has always been this way or if my sister and I had to do something with it…… That, I don’t know……./T: And your sister, does she asks such questions?/P: I don’t know./T: You don’t talk about that?/P: No, we don’t talk about that.

What we see here is that the therapist keeps insisting, despite Pam’s very short and dismissive answers. Expressive interventions remain high up until session 12, with the exception of session 4 and session 7. In these sessions, there are less expressive interventions because the therapy session mainly focuses on Pam receiving a negative evaluation on her job (session 4) and losing her job (session 7). The therapist uses supportive techniques to convey an empathic understanding towards her (e.g. ‘I notice it is hard on you.’; ‘I’m really sorry for you.’). Expressive interventions during these sessions continue to focus on Pam’s main interpersonal difficulty, namely being unable to open up to others. Interventions specifically aim at elaborating this issue. The intervention ‘Did you talk to anyone about that?’, for instance, appears multiple times in all sessions and are always followed by naysay. Therapy session 6 is an exemption in this regard:

T: You say ‘they still try to control me.’ It strikes me that you don’t let anyone control you, very persistently./P: Yes. Maybe I’m too controlling. That is perhaps the sore point./T: I’m thinking about you not informing anyone about the epilepsy. It sounds as if you don’t want anyone to influence or control your decisions./P: Yes, that might be./T: Or do you see it differently?/P: No, what you say is right ……… I want to be my own boss./T: Yes, that is something I heard you say a couple of times, but it also seems – how do I say this – a lonely position./P: Yes, that is the down side. Maybe that is why I’m so unhappy, because I’m lonely./T: Are you lonely?/P: I think so./T: You don’t share a lot with people./P: I always see dangers on the road. I’ve been hurt by people I confided in too many times and they used that against me.

Here, Pam recognizes and marks some delicate interpersonal core issues. In session 8, which is the sessions with the highest absolute number of expressive interventions, she further elaborates on not being able to open up to others. She now expresses a wish to change that.

P: It is always tough to let other people in. I want to change that and start confiding in my sister more. Last Saturday, I had the chance to say I no longer have a job, but then I just don’t say that./T: You remember what stopped you? How would she have reacted?/P: I was just waiting for the right moment and then I dropped her off and I hadn’t said it. I wanted to./T: Speaking up is important. If you stop talking, it has an effect./P: Yes, that is starting to dawn on me./T: You stopped talking at a very young age at home, but at a sudden point also outside something stopped./P: Yes, in a variety of ways my speech has flattened. To just call on someone or say something about myself. That does not run smoothly. To learn that a bit, my sister might be the most convenient person to take the first steps./T: You think you should learn that now?/P: Yes, I think so. I think it is time to change.

Up until session 11, the expressive interventions continue to explore, elaborate and try to work through these issues of trust and being unable to open up. Sessions 9 and 11 were followed by epileptic insults, which Pam linked to the intensified stress she experienced in these therapy sessions. After session 11, the therapist received an email from Pam in which she expressed doubt about continuing therapy and not wanting to bother the therapist any further. Before session 12, the therapist voiced her concerns about this case in an intensive supervision session. She wondered about whether or not it was her own desire to let Pam work through issues concerning her childhood traumas and the relationship with her mother and if the therapy should take another turn in order to help Pam to feel better rather than worse. The conclusion of the supervision session was that the therapist perhaps should not insist on elaborating these difficult issues, especially when Pam would show bodily signs of stress. Moreover, it was proposed that the therapist could work together with her patient to find words for what her body was trying to say. From session 12 onwards, we see that the number of supportive interventions rises exponentially.

T: I think it is really important that you can talk to someone. I understand how hard it is for you to open up about the past. Perhaps we should not avoid it completely because, in any way, you and your history are interconnected, but perhaps we should take things a bit more slowly. Opening up can only happen in a safe environment.

With these supportive interventions, the therapist emphasizes having heard Pam’s message and that she recognizes the profound impact therapy has on her. She encourages Pam to continue treatment whilst also allowing her agency in treatment and showing respect for her boundaries and decisions. In the following sessions, we also see a shift in the themes that are discussed. Issues of bodily symptoms and difficulties in current relationships are now more on the forefront. Moreover, the therapist often steers the conversation away from the more intimate topics when she or Pam recognize an increase in stress reactions to more safe issues, such as day-to-day schedules and more long-term plans. Next to that, it is noticeable that when intimate topics are discussed, the therapist is less persevering and more cautious in delivering certain messages, by building an expressive message on a supportive foundation.

T: Tension makes the body cramp, a tension that arises from a fear, an anxious feeling and to what that is connected, perhaps we can go into that. We can take our time to do that./P: Yes. I know that when my parents come, I panic. But I don’t believe that I’m very tense once they are inside. Although, maybe. I don’t really know………/ T: Do you sometimes relax your body?/P: Not really. When I go to bed, then perhaps./T: Well, that’s something we should not shy away from. Also here, when you talk. If I notice something, is it okay I say something about it or is that inappropriate?/P: No, that’s okay. I can’t do much about it anyway./ T: No, of course you can’t. That’s also not what’s at stake here, but I think we should consider it, because your body also speaks, whether or not we immediately know what it says. Over time, we’ll figure out why that is or what it is connected to. (session 14)

In the last four sessions, the therapist inquires several times about whether or not Pam would want to continue treatment after the assigned 20 sessions in the context of the GPS study and what she would like to talk about in those last sessions. In session 18, Pam indicates that her depressive symptoms are lessening and that she is still in doubt about whether or not to continue treatment afterwards. Here, the therapist supports the progress Pam made over the course of therapy. On the other hand, she draws attention to the deeper-rooted destabilizing influence the relationship with her parents might have. Pam recognizes what the therapist is saying and indicates that while speaking up in treatment feels *no longer* unsafe, she still experiences troubles outside the therapy room. However, because she feels better now, she does not know if she wants to explore things further. In session 19, however, there was significant work done concerning the relationship with her parents. Here, we see that Pam could enunciate important questions about her upbringing, whereas at the beginning of treatment she was very reluctant to do so. Nevertheless, session 20 takes a radically different turn. Pam enters the session with great news: she was selected for a job and was very excited. The therapist echoes Pam’s enthusiasm and confirms that having a job and daily structure were important themes throughout the sessions. She repeats the question about whether Pam would like to continue working around the subject of her parents or if she would rather close the subject down. Pam indicates that questions about that topic specifically surface during but not outside their sessions and suggests she would reconsider the offer to come back if questions would arise outside the therapy room as well. The therapist suggest they leave it at that and ends with firmly expressing her commitment to continue their work in the future if and when a new request for therapy would arise.

## Discussion

The first and second aim of this study was to investigate the nature of interpersonal patterns at the beginning and throughout treatment. We found that Pam experienced a strong inability to open up, which could be traced back to the relationship with her parents, whom were always very critical towards her. This resulted in the feared anticipation of rejection in later relationships, both at the level of love and work. Pam strove to avoid such confrontations by keeping silent. As treatment progressed, we learned that the parental disdain also involved a lack of valuing Pam and being interested in her, which she also encountered in her adult relationships. At the end of treatment, Pam clearly expressed a desire for others to take a genuine interest in her. Further, she conveyed the wish to be able to communicate openly with close relatives. Although Pam communicates the wish to be close to others, as articulated in the wish to be liked and to be respected, she does not show any action towards achieving those goals. Instead, she persistently upholds a passive demeanour because she anticipates disappointment. Moreover, Pam does not articulate the wish to be distant from others as such. She rather expresses a wish to avoid conflict and not to be hurt, i.e., to not be confronted with the anticipated criticism and rejection. It is our contention that the wish to avoid conflict actually has nothing to do with favouring distance, but that also here, the underlying wish is to be genuinely close to others. From an attachment perspective, this observation converges with Bowbly’s postulation that it is the fundamental human condition to need proximity (Waldinger et al., 2003). It begs the question whether the wish to be close to others is a unique component of the interpersonal dynamics associated with complex trauma or rather a basic feature of human desire. In the broader field of studies concerning interpersonal patterns related to psychopathology, it has been found that the most common wish is to be close to others and to be accepted (Wilczek, Weinryb, Barber, & Gustavsson, 2010).

The negative (anticipated) reactions from others and Pam herself do not change over time, which is also reflected in the stagnating IIP-32 scores. However, Pam was able to communicate her desire for close relations more openly as treatment progressed. The lack of change in the perceived reactions of others and her own interpersonal behaviour shows how difficult deeply engrained interpersonal patterns are to transform (e.g., Pearlman & Courtois, 2005) and that short-term treatment might not suffice to achieve change. Nevertheless, the follow-up data suggest that the treatment did commence a process of change, of which the therapeutic effects were only visible as time progressed (Leichsenring & Schauenburg, 2014).

The third and final aim of this study was to investigate the therapy process, by mapping the therapeutic relationship and therapist interventions. We saw that Pam initially reported a worsening of her condition, which she strongly linked to rising levels of stress both outside (e.g., impending unemployment) and inside the therapy room. The therapist used a large number of expressive interventions, specifically aimed at exploring the traumatic relationship between Pam and her parents. Before discussing the case in supervision, she kept insisting on analysing these matters, notwithstanding Pam’s reluctant stance, which was obvious from her short and resistant answers (e.g. ‘I don’t know’). This phase in the therapy process – which lasted up until session 11 – bears resemblance to treatment modalities that straightforwardly focus on the traumatic contents (e.g. Wagenmans et al., 2008). After supervision, the therapist applied a different strategy, by focusing more on current difficulties and applying more supportive interventions. Pam responded well to the change in focus, which was demonstrated by symptom improvement. However, there were some unresolved interpersonal issues. As we have seen, the therapist alluded to the possibility of working through these difficulties in continued treatment. She did not force this on Pam, but rather informed her, communicated her commitment and willingness to continue their work together, and left the choice up to her. Pam did not take up this proposal, but always kept the possibility in mind if these or other issues would impede her daily functioning.

These observations show the importance of allowing patients agency in therapy (Lawson et al., 2013). Further, our results illustrate the importance of being aware of the impact interventions have on patients and that therapists should reappraise their approach if necessary (Stiles, 1998). Supervision can help clinicians to address these issues (Pearlman & Courtois, 2005).

Given Pam’s levels of distress, more supportive techniques were favoured, which is in line with treatment modalities focusing on stabilization (e.g., Classen et al., 2017). However, the results of the WAI-SR did not support the underlying reasoning behind the need for stabilization, namely that a trusting relationship in the therapy would be difficult to establish. This might imply that no special consideration should be given to building and sustaining a safe therapeutic relationship in this case. However, based on our qualitative analysis, we deem it necessary to consider alternative explanations. From the CCRT analysis, we learned that Pam views others as untrustworthy and critical, which causes her to be rigidly introverted and apprehensive in interactions. From developmental and attachment theories, we would expect these issues to resonate in the treatment context (Ebert & Dyck, 2004; Pearlman & Courtois, 2005). There were several occasions in which the dominant CCRT components transpired in the therapeutic context, for instance when Pam stressed the professionalism of the therapist or said that talking in therapy was safer and easier because the therapist was in no position to pass down information to her parents. This remarkable comment suggests that it was the therapist’s confidentiality obligations that prohibited a repetition of what she would normally expect. It thus seems that Pam’s remark roots from the same dominant patterns that structure her interpersonal interactions. Although Pam *knows* she is safe on the basis of the professional duties of the therapist, a fundamental *feeling* of trust or a sustainable and intrinsic experience of the therapeutic context as a safe environment seems lacking. These observations show the perseverance of dominant interactional patterns (Luborsky, 1986), how their repetitive nature affects the therapeutic encounters (Ebert & Dyck, 2004; Gleiser et al., 2008), but also how Pam remains unaware of the influence these patterns have on her stance in therapy. This raises the question whether the WAI-SR is able to capture the underlying dynamics in therapy. It appears that Pam filled in the WAI-SR based on her rational knowledge about the therapeutic setting, yet that her answers were not indicative for her inner experiences. This suggests that the WAI-SR scores should not be taken at face value and should always be considered within the broader narrative of the patient (Desmet, 2018).

Just as self-report measures might not always capture ‘the full story’, therapists have the reputation of being poor judges of patients’ well-being (e.g., Dimidijian & Hollon, 2010; Hatfield et al., 2010). Also in Pam’s case, the therapist initially seemed unable to make a fair estimation of Pam’s condition. By reviewing her case in supervision, however, she recognized the deteriorating effect the therapy produced. This stimulated a fundamental change in the therapeutic bond. For instance, the therapist now noticed Pam’s distress and acknowledged it explicitly in therapy. Further, she started using more supportive techniques by which she conveyed her commitment and a genuine interest. She commended Pam for expressing herself in treatment and did not reprimand her or gave advise about the choices she made. Finally, Pam commented that, whereas opening up to others remained troublesome, speaking up in therapy no longer felt unsafe. All these considerations suggest that the therapist provided a new relational experience for Pam (e.g. Lawson et al., 2015) in which, eventually, she could open up more safely. The use of supportive interventions will definitely have played an important role in creating a safe atmosphere. However, Pam’s case also shows that general interventions might serve the same purpose. Specifically interesting here is Pam’s remark about the therapist asking questions, in contrast to people in her environment, whom would not ask any questions at all. Against this background, the large number of general interventions appear to have had another function in the treatment process than merely keeping the conversation going. That is, by asking (neutral) questions, the therapist conveyed a genuine interest in Pam, which contributed to the creation of a new relational experience. What we deduce from this is that treatment interventions must always be considered in the context of the effects they produce in a particular case (Stiles, 2013). This further shows the importance of challenging habitual therapy practices and considering alternative views on the treatment process, especially, but not exclusively, when the therapy process is stagnating or produces negative effects.

There were some remarkable discrepancies between the different qualitative and quantitative measures, which require some further comments. First, Pam did not receive a diagnosis of PTSD as assessed during the pre-treatment interviews, whereas her ZIL-score suggest she suffered from typical PTSD-symptoms, such as hyperarousal and avoidance. The ZIL only assesses symptom severity and not traumatic antecedents. When asked for traumatic experiences during the PTSD-module of the SCID-I interview, Pam did not mention her childhood experiences, nor other traumatic experiences that caused continued suffering. Therefore, this module was terminated. The SCID-I interview mainly assesses recent, acute and single-incident traumatic events. This can explain why Pam failed to mention the exposure to past and chronic childhood traumatic experiences. Another possible explanation is that Pam avoided to talk about her upbringing in-depth or that, at that moment in time, she did not connect her suffering to the traumatic relationship with her parents. In the literature, it has been widely acknowledged that the psychological consequences connected to traumatic experiences can be very diverse and that co-morbid conditions, such as depression, can be communicated more explicitly when seeking treatment (e.g., Van der Kolk et al., 2005). This shows the importance of more clinically oriented intake procedures. We advocate a case formulation approach in order to shift the emphasis from merely inventorying complaints and symptoms to the inclusion of the broader (psychological) context and experiences of the patient (Eells, 2007; Vanheule, 2017).

It should also be noted that there is an important inconsistency between the self-report outcome questionnaire scores and Pam’s narrative concerning therapy outcome. Whereas the outcome measures suggest no improvement or even deterioration, the qualitative data indicates otherwise and suggests depressive symptoms have significantly declined. These irregularities further show the importance of triangulation (Jackson et al., 2011) and complementing quantitative findings with narrative information (Desmet, 2018). Our findings demonstrate that the use of single measures can lead to inconsistent findings, which might lead to ambiguous conclusions. Therefore, a mixed-methods approach is recommended to advance the field of therapy research, as the limitations of both quantitative and qualitative measures and techniques can be compensated through the complementary use of both (Dattillio, Edwards, & Fishman, 2010).

Next to the methodological limitations regarding the interpretability of certain self-report outcome and process measures, we need to address the restrictions associated with single-case research, especially with regards to the generalizability and transferability to other cases (Levitt et al., 2018). As our aim was to deepen our understanding of interpersonal features in complex trauma (i.e., enriching, Stiles, 2013), we selected a case based on criteria that invoke rich information. In retrospect, Pam can also be considered a critical case (Patton, 2002), on account of the intricate interconnections between Pam’s core interpersonal patterns and the formation of the therapeutic relationship, which was demonstrated by the erratic sequence of supportive and expressive interventions. Our results necessitated to refine certain theoretical assumptions (i.e., theory-building, Stiles, 2013) and provided some interesting insights with regards to the influence of core interpersonal patterns on the therapy process. What we distilled from Pam’s case is that therapists should be aware that patients’ dominant interpersonal schemes slip into the therapeutic relationship, sometimes in very subtle ways. Therefore, sufficient attention should also be paid to the discrepancies between what patients rationally acknowledge and the underlying impulses which might unconsciously affect the therapeutic relationship. If it turns out that a constitutive feeling of trust is lacking or has not yet been appropriately established, then, the therapist should adjust his or her therapeutic approach accordingly (e.g., via additional supportive techniques) and search for ways to allow for a new relational experience for the patient.

Our preliminary conclusions can inspire several avenues for further research. Specific for patients with a history of trauma, it would be interesting to study the formation of the therapeutic relationship and broader changes in interpersonal dynamics in patients with overt issues of trust. Next to that, it would be interesting to study the process of change in patients who do not readily connect their suffering to their trauma background. More generally, our results compel more research into the effects of dominant interpersonal patterns on the formation of the therapeutic relationship and the therapeutic process. Further, therapists’ implementation of interventions and therapist responsiveness remain unexplored territory. Ultimately, having more detailed and multi-angled knowledge of the mechanisms of change in therapy can lead to increasingly focused and differentiated treatment goals and guidelines and dynamically give shape to the treatment process.
